# Prevalence, incidence and predictors of renal impairment in persons with HIV receiving protease-inhibitors in rural Tanzania

**DOI:** 10.1371/journal.pone.0261367

**Published:** 2021-12-15

**Authors:** Herry Mapesi, James Okuma, Fabian Franzeck, Herieth Ismael Wilson, Elizabeth Senkoro, Theonestina Byakuzana, Robert Ndege, Fiona Vanobberghen, Tracy Renée Glass, Manuel Battegay, Maja Weisser, Daniel Henry Paris, on behalf of the KIULARCO Study Group

**Affiliations:** 1 Ifakara Health Institute, Ifakara Branch, Ifakara, United Republic of Tanzania; 2 Swiss Tropical and Public Health Institute, Basel, Switzerland; 3 University of Basel, Basel, Switzerland; 4 St. Francis Referral Hospital, Ifakara, United Republic of Tanzania; 5 Division of Infectious Diseases and Hospital Epidemiology, University Hospital Basel, Basel, Switzerland; University of Cape Town, SOUTH AFRICA

## Abstract

**Objective:**

Ritonavir-boosted protease inhibitors (bPI) in people living with HIV (PLWH) have been associated with renal impairment. Limited data are available from rural sub-Saharan Africa.

**Methods:**

Using data from the Kilombero and Ulanga Antiretroviral Cohort Study (KIULARCO) in rural Tanzania from 2005-01/2020, we assessed the prevalence of renal impairment (estimated glomerular filtration rate <60 mL/min/1.73m2) at the time of switch from first-line antiretroviral treatment (ART) to bPI-regimen and the incidence of renal impairment on bPI. We assessed risk factors for renal impairment using logistic and Cox regression models.

**Results:**

Renal impairment was present in 52/687 PLWH (7.6%) at the switch to bPI. Among 556 participants with normal kidney function at switch, 41 (7.4%) developed renal impairment after a median time of 3.5 (IQR 1.6–5.1) years (incidence 22/1,000 person-years (95%CI 16.1–29.8)). Factors associated with renal impairment at switch were older age (adjusted odds ratio (aOR) 1.55 per 10 years; 95%CI 1.15–2.11), body mass index (BMI) <18.5 kg/m^2^ (aOR 2.80 versus ≥18kg/m^2^; 95%CI 1.28–6.14) and arterial hypertension (aOR 2.33; 95%CI 1.03–5.28). The risk of renal impairment was lower with increased duration of ART use (aOR 0.78 per one-year increase; 95%CI 0.67–0.91). The renal impairment incidence under bPI was associated with older age (adjusted hazard ratio 2.01 per 10 years; 95%CI 1.46–2.78).

**Conclusions:**

In PLWH in rural sub-Saharan Africa, prevalence and incidence of renal impairment among those who were switched from first-line to bPI-regimens were high. We found associations between renal impairment and older age, arterial hypertension, low BMI and time on ART.

## Introduction

Rollout and improvement in HIV care and treatment have led to a shift in the main causes of morbidity and mortality in people living with HIV (PLWH) away from opportunistic infections to chronic non-communicable diseases such as liver, cardiovascular and renal diseases [1]. With a global prevalence of 11–13% [2], chronic kidney disease (CKD) is regarded as an independent risk factor for cardiovascular diseases (CVD) and a leading cause of mortality and morbidity in PLWH [3, 4].

The pooled prevalence of CKD among PLWH in sub-Saharan Africa (sSA) is estimated to be 14.6% [5]. Apart from traditional risk factors for CKD such as arterial hypertension and diabetes mellitus, PLWH in sSA are facing the additional burden of other infectious diseases associated with CKD and renal impairment such as tuberculosis and schistosomiasis [6]. In Tanzania, the prevalence of CKD in the general population is estimated to be around 7–15% and in PLWH up to 28% [6–9].

Among PLWH, long-term toxicity of antiretroviral treatment (ART) remains a key concern. World Health Organization (WHO) treatment guidelines recommend the use of tenofovir disoproxil fumarate (TDF) as first-line nucleotide reverse transcriptase inhibitor (NRTI) in combination with either emtricitabine (FTC) or lamivudine (3TC) and a non-nucleotide reverse transcriptase inhibitor (NNRTI) or an integrase inhibitor [10]. For second-line treatment, the same guidelines recommend the use of TDF in combination with ritonavir-boosted protease inhibitors (bPI)—either atazanavir (ATV/r) or lopinavir (LPV/r). The Tanzania treatment guidelines follow the WHO treatment guidelines [11]. Longer cumulative exposure to the use of TDF [12, 13] and bPIs [14, 15] has been associated with CKD or kidney dysfunction. Furthermore, the risk of kidney dysfunction increases when patients receive TDF in combination with a bPI [15–17]. The mechanism of CKD is not well understood, although previous studies suggested it might be due to crystalluria, proximal tubular dysfunction, urolithiasis, and interstitial nephritis [18, 19].

Using data from a cohort of HIV-positive patients in rural Tanzania—the Kilombero and Ulanga Antiretroviral Cohort (KIULARCO)—we investigated the prevalence and associated factors of renal impairment in PLWH at the time of switch from NNRTI to bPI, and renal impairment incidence and associated factors among those who had normal kidney function at the time of switch from NNRTI to bPI.

## Methods

### Study design and settings

The KIULARCO is a prospective cohort with more than 11,000 PLWH ever enrolled and 4,500 on active follow-up as of January 2020. Since 2005, all individuals who attend the Chronic Diseases Clinic of Ifakara (CDCI) at the St. Francis Referral Hospital in Ifakara, Tanzania are asked for consent to participate in the cohort. Data on demographics, clinical presentation, ART, HIV-1 RNA viral load, CD4 T-cell count and other laboratory investigations are recorded electronically at enrolment and during follow-up visits. Details of the cohort have been published elsewhere [20, 21].

In this nested study, participants from KIULARCO who were switched from first-line ART regimens to bPI-based regimens were eligible, if they met the following criteria: age ≥15 years, availability of eGFR measurement within 6 months before or after the time of the ART switch and at least one eGFR measurement during follow-up visits. We excluded participants who either started treatment with regimens containing a bPI or entered the cohort while receiving bPI. All participants were offered treatment and care according to the Tanzania standard treatment guidelines [11].

### Procedures

We included participants who attended KIULARCO from 2005 to January 2020. During the time of the study, participants started ART with at least 2 NRTIs and 1 NNRTI. The use of Integrase strand transfer inhibitor (dolutegravir) as first-line ART was implemented only in March 2019 [11]. By the time of this analysis, no participant had been switched from dolutegravir to bPI, therefore participants on dolutegravir were not included in this analysis. During follow-up, once a clinician suspected clinical or immunological failure, participants were switched to second-line that usually contained 2 NRTIs and a bPI [11]. Routine HIV-1 RNA viral load monitoring was not available until 2018, thus, treatment failures mostly were not confirmed virologically.

### Laboratory and clinical investigations

Routine laboratory investigations (serum creatinine, CD4 cell count, aspartate aminotransferase, alanine aminotransferase and complete blood cell count) were performed routinely once a year. Additional measurements can be performed upon clinical indication. During the time of the study, routine HIV-1 RNA viral load monitoring was not available, however, it was performed upon physician request if immunological or treatment failure was suspected or if participants were enrolled in a specific nested study within a cohort. For all laboratory and clinical measurements, we considered the closest measurement prior to the switching date from NNRTI to bPI within the time window of six months before or after the switch. We measured serum creatinine using Cobas c 111 Analyzer (Roche Diagnostics, Rotkreuz, Switzerland) and we used the Chronic Kidney Diseases Epidemiology (CKD-EPI) formula to calculate the estimated glomerular filtration rate (eGFR) [22]. We measured the CD4 cell count using flow cytometry (BD FACS Calibur, Franklin Lakes, NJ) and categorized into CD4 cell counts <200 cell/mm3 or ≥200 cells/mm^3^ [23].

We defined tuberculosis (TB) if the participant had a recorded positive GeneXpert (Cepheid, Sunnyvale, CA) result in the sputum or another body fluid sample. The GeneXpert was established in KIULARCO from 2013 onwards. Additionally, participants were categorized to have TB if they had a chest x-ray suggesting TB with at least 1 TB symptom and receiving TB treatment at the time of switch.

Blood pressure and body weight were measured at every clinical visit. We defined arterial hypertension as having either a systolic BP of ≥140mmHg and/or a diastolic BP ≥90mmHg on 2 consecutive clinical visits [24], being currently on antihypertensive treatment or a previous history of arterial hypertension diagnosis. Body mass index (BMI) was calculated using weight (kilograms/height^2^ (m^2^)) and was categorized as either underweight (BMI <18.5 kg/m^2^), normal weight (18.5–24.9 kg/m^2^) or overweight (≥25 kg/m^2^). The current ART regimen before the switch (pre-bPI) was defined as the closest ART regimen the patient was receiving before the switch. The bPI (post-bPI) was defined as the first second line ART the patient received after switch.

### Statistical analysis

We extracted demographic, clinical, laboratory and treatment information from the KIULARCO electronic database. We summarized categorical variables using frequencies and percentages and continuous variables using median and interquartile range (IQR). We used logistic regression models to assess the predictors of renal impairment at the time of switch from NNRTI to bPIs. Results are presented with odds ratios (OR) and 95% confidence intervals (CI).

Cox regression models were fitted to assess the association between covariates at the time of the switch to bPIs and development of renal impairment. Study participants who had renal impairment at the time of switch to bPI were excluded from this analysis. Results are presented with hazard ratios (HR) and 95% CI.

Renal impairment was defined as having moderate or severe eGFR decrease (<60 mL/min/1.73m2) at the time of the switch from first-line ART to bPI by using single eGFR measurement (Kidney Diseases Improving Global Outcomes KDIGO Stage G3-G5) [25]. Additionally, for participants who had normal kidney function at the time of the switch we defined the incidence of renal impairment if their eGFR dropped to <60 mL/min/1.73m2 using single eGFR measurement [25]. The differentiation of renal impairment into CKD and acute kidney disease (AKI) was not possible due to lack of systematic repeat creatinine measurements in routine care [26]. In most of the studies from sSA estimation of renal impairment is based on one measurement only due to lack of repeat measurements of serum creatinine [6, 27–29]. We conducted a sensitivity analysis to assess CKD for participants who had two measurements of eGFR <60 mL/min/1.73m2 three months as recommended by KDIGO [25].

A priori, we identified the following variables for multivariable analysis: age, BMI, HIV World Health Organization (WHO) stage, CD4 cell counts, hypertension, ART regimen, calendar year of switching, and TB. All analyses were performed using Stata, version 15.1 (Stat Corp, College Station, Texas, USA).

### Ethics statement

All patients’ data were anonymized before we conducted analysis. At enrolment in KIULARCO, a written informed consent is sought from all study participants; those who refused consent were excluded. Ethical approval was obtained from the Ifakara Health Institute review board (IHI/IRB/No16-2006), the National Health Research Committee of the National Institute of Medical Research of Tanzania (NIMR/HQ/R.8a/Vol.IX/620) with yearly renewal, as well as from the Ethikkomission Nordwest und Zentralschweiz (EKNZ; Switzerland).

## Results

**Fig 1:**
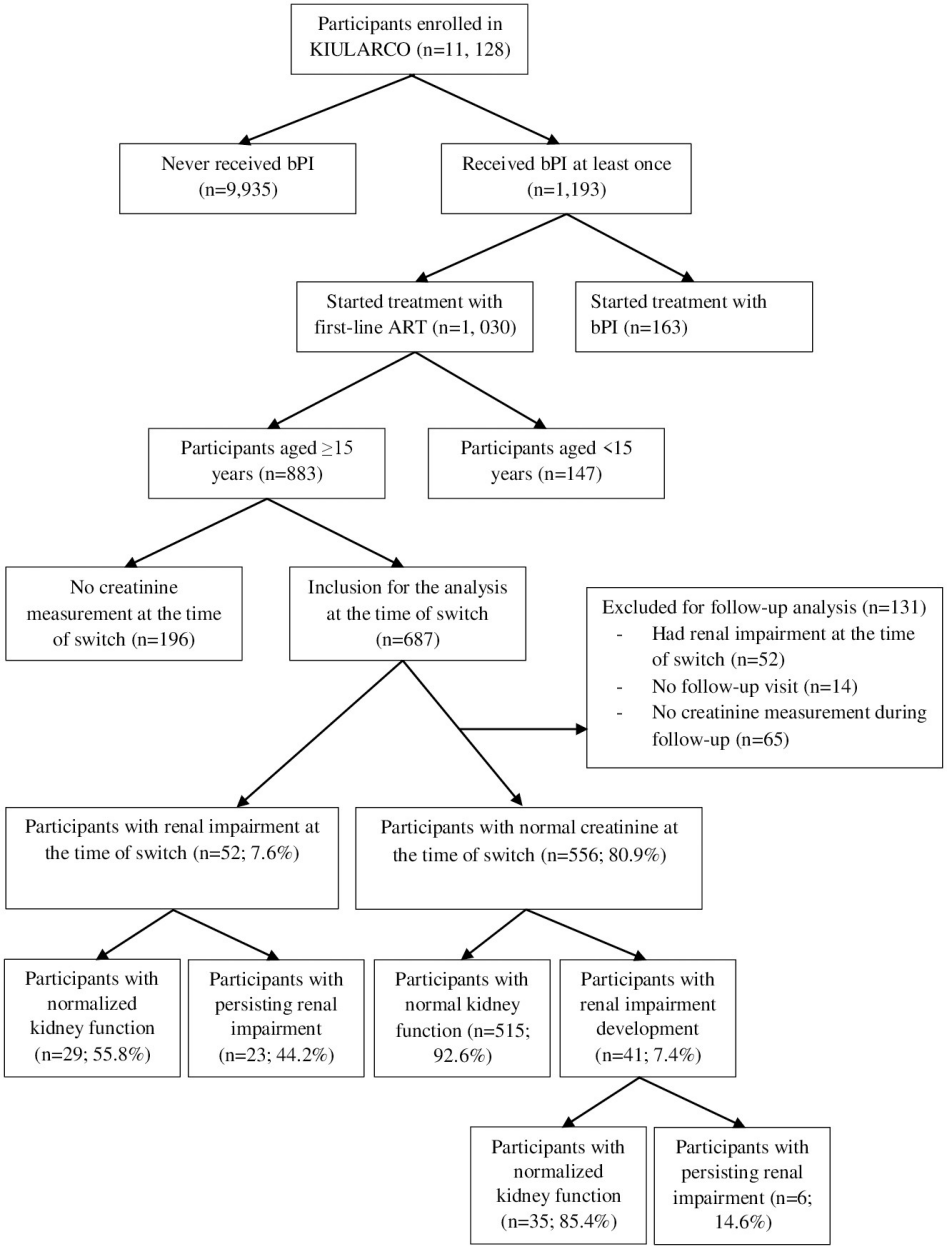
Participants’ flow chart.

A total of 11,128 participants were enrolled into KIULARCO from 2005 up until January 17, 2020. Of these, 9,935 were never prescribed a bPI-regimen and 163 participants were excluded because they started treatment with a bPI-regimen at the time of enrolment or entered the cohort already receiving a bPI-regimen. A further 147 participants were aged <15 years, and 196 did not have creatinine measurements at the time of switch. For this analysis, 687 participants were included at the time of switch to a bPI-regimen, of whom 556 had a normal baseline creatinine and at least one follow-up creatinine measurement (Fig 1).

Demographic and clinical characteristics of participants at the time of switch from first-line ART to bPI-regimen are summarized in Table 1. The majority of participants (63.5%) were female, with a median age of 41 years (IQR 33–48). An advanced WHO stage III or IV was present in 60.7% of participants. The median BMI was 21.68 kg/m^2^ (IQR 19.1–24.5) with 53% of participants having a normal BMI (≥18.5–24.9 kg/m^2^). The median CD4 cell count was 218 cells/mm^3^ (IQR 95–383), with 52.1% of participants having a CD4 cell count of <200 cells/mm^3^. There were 61 participants (8.9%) who were diagnosed with tuberculosis and 116 (16.9%) were diagnosed with arterial hypertension either 6 months before or after switch.

**Table 1:** Characteristics of the 687 patients at the time of switch to second-line treatment.

	Total	Normal kidney function at baseline (eGFR ≥60 ml/min/1.73m^2^)	Renal impairment at baseline (eGFR <60 ml/min/1.73m^2^)
	n = 687	n = 635	n = 52
Sex			
Male	251 (36.5)	234 (36.9)	17 (32.7)
Female	436 (63.5)	401 (63.2)	35 (67.3)
Age, years, Median (IQR)	40.9 (33.17–48.48)	40.8 (32.5–48.3)	43.7 (36.2–57.2)
WHO Stage			
I	119 (17.3)	116 (18.3)	3 (5.8)
II	145 (21.1)	136 (21.4)	9 (17.3)
III	234 (34.1)	219 (34.5)	15 (28.9)
IV	183 (26.6)	161 (25.4)	22 (42.3)
Missing	6 (0.9)	3 (0.5)	3 (5.8)
BMI (Kg/M^2^)			
<18.5	115 (16.7)	97 (15.3)	18 (34.6)
≥18.5–24.9	364 (53.0)	341 (53.7)	23 (44.2)
≥25	140 (20.4)	133 (20.9)	7 (13.5)
Missing	68 (10.0)	64 (10.1)	4 (7.7)
CD4 cell count (cells/mm3)			
<200	358 (52.1)	260 (40.9)	30 (57.7)
≥200	290 (42.2)	342 (53.9)	16 (30.8)
Missing	39 (5.7)	33 (5.2)	6 (11.5)
Tuberculosis			
Yes	61 (8.9)	52 (8.2)	9 (17.3)
No	623 (90.7)	581 (91.5)	42 (80.8)
Missing	3 (0.4)	2 (0.3)	1 (1.9)
Arterial hypertension			
Yes	116 (16.9)	101 (15.9)	15 (28.9)
No	560 (81.5)	527 (83.0)	33 (63.5)
Missing	11 1.6)	7 (1.1)	4 (7.7)
ART before switch			
AZT+3TC+NVP	145 (21.1)	139 (21.9)	6 (11.5)
AZT+3TC+EFV	93 (13.5)	90 (14.2)	3 (5.8)
TDF+FTC+EFV	90 (13.1)	81 (12.8)	9 (17.3)
TDF+FTC+NVP	26 (3.8)	23 (3.6)	3 (5.8)
TDF+3TC+EFV	302 (44.0)	284 (44.7)	18 (34.6)
ABC+3TC+EFV	24 (3.5)	13 (2.0)	11 (21.2)
Other first-line ART	7 (1.0)	5 (0.8)	2 (3.8)
Viral load (copies/ml)*			
<1000	252 (36.7)	238 (37.5)	14 (26.9)
≥1000	141 (20.5)	131 (20.6)	10 (19.2)
Missing	294 (42.8)	266 (41.9)	28 (53.9)
Time on ART before switch (years)			
Median (IQR)	3.6 (1.5–6.0)	3.7 (1.6–6.1)	1.48 (0.6–4.2)
Calendar year of switch			
<2015	363 (52.8)	338 (53.2)	25 (48.1)
≥2015	324 (47.2)	297 (46.8)	27 (51.9)

Abbreviations: eGFR, estimated glomerular filtration rate; BMI, body mass index; WHO, World Health Organization; IQR, inter quartile range; ART, antiretroviral treatment; AZT, zidovudine; 3TC, lamivudine; NVP, nevirapine; EFV, efavirenz; TDF, tenofovir disoproxil fumarate; FTC, emtricitabine; ABC, abacavir.

*requested only in patients with suspected treatment failure and those who were involved in s specific study

The median time on first-line ART before the switch to bPI-based regimen was 3.6 years (1.5–6.0); the most common first-line treatment used at the time of switch was TDF in combination with lamivudine (3TC) and efavirenz (EFV) (44.0%), followed by zidovudine (AZT), 3TC and nevirapine (21.1%), AZT, 3TC and EFV (13.5%) and TDF, emtricitabine (FTC) and EFV (13.1%). Majority of participants 363 (52.8%) were switched to bPI before the year 2015.

### Renal impairment at the time of switch

The median eGFR at the time of switch was 126.2 ml/1.73m^2^ (IQR 102.9–40.8). A total of 52 participants (7.6%) had renal impairment at the time of switch. In these participants, renal impairment resolved (eGFR ≥60 mL/min/1.73m2) in 29 (55.8%) by the time of study closure, therefore, most likely AKI.

Results from univariable and multivariable logistic regression models assessing the association of renal impairment and independent predictors at the time of switch are presented in Table 2. After adjustment for potential confounding factors, we found evidence of associations between renal impairment and increasing age (adjusted odd ratio (aOR) 1.55 per 10 years; 95% CI 1.15–2.11; *P* 0.004), BMI <18.5 kg/m^2^ (aOR 2.80 versus ≥18.5 kg/m^2^; 95% CI 1.28–6.14; *P* 0.010) and arterial hypertension (aOR 2.33; 95% CI 1.03–5.28; *P* 0.043). The risk of renal impairment was reduced with increased exposure to first-line ART (per one-year increase) (aOR 0.78; 95% CI 0.67–0.91; *P* 0.002). We did not find an association between renal impairment and CD4 cell count, WHO stage, TB diagnosis, and calendar year of switch.

**Table 2:** Univariate and multivariate logistic regression model for predictors of renal impairment at switch to second line ART (n = 687).

Variable	Univariate model Unadjusted OR (95% CI)	P value	Multivariate model Adjusted OR (95% CI)	P value
Age (per 10 years)	1.43 (1.15–1.79)	0.002	1.55 (1.15–2.11)	0.004
BMI (Kg/M^2^)				
≥18.5	1			
<18.5	2.84 (1.53–5.28)	0.001	2.80 (1.28–6.14)	0.010
CD4 count (cells/mm^3^)				
≥200	1		1	
<200	2.47 (1.32–4.62)	0.005	1.29 (0.63–2.66)	0.492
WHO stage				
I or II	1		1	
II or IV	2.04 (1.05–4.00)	0.036	1.29 (0.59–2.84)	0.520
Arterial hypertension				
No	1		1	
Yes	2.37 (1.24–4.53)	0.009	2.33 (1.03–5.28)	0.043
Tuberculosis diagnosis				
No	1		1	
Yes	2.39 (1.10–5.19)	0.027	2.11 (0.86–5.20)	0.103
Total time on ART before the switch (per one year)	0.81 (0.72–0.92)	0.001	0.78 (0.67–0.91)	0.002
Calendar year of switch				
<2015	1		1	
≥2015	1.22 (0.70–2.16)	0.475	1.17 (0.59–2.31)	0.650

Abbreviations: BMI, body mass index; ART, antiretroviral treatment; OR, odds ratio; CI, confidence interval; WHO, World Health Organization

### Renal impairment during follow-up

The median time under observation for the 556 participants who had normal renal function at the time of switch and at least one follow-up creatinine measurement was 3.49 years (IQR 1.63–5.01) (S1 Table). The majority of participants (161 participants, 29%) received an ATV/r-based regimen (TDF, FTC, ATV/r, 29%) followed by TDF, FTC and LPV/r (113 participants, 20.3%).

A total of 41 (7.4%) participants developed renal impairment during follow-up at the median time of 3.5 years (IQR 1.6–5.1). Among these participants, renal impairment resolved (eGFR ≥60 mL/min/1.73m2) in 35 (85.4%) by the time of study closure. The total time at risk was 1,864.10 person-years and the incidence of renal impairment was 22 per 1,000 person-years (16.1–29.8). Results from univariable and multivariable Cox regression models assessing the association of development of a new renal impairment under bPI treatment and independent predictors at the time of switch are presented in Table 3. We found strong associations between development of renal impairment and older age (adjusted hazard ratio (aHR) 2.22 per 10 years; 95% CI 1.56–3.15; *P* <0.001). We found some association between development of renal impairment and being switched to a TDF-based regimen (aHR 2.12 versus non-TDF-based regimen; 95% CI 0.93–4.80; *P* 0.072). We did not find evidence of an association between development of renal impairment and BMI (aHR 0.96 for <18 versus ≥18 kg/m^2^; 95% CI 0.37–2.43; *P* 0.924), CD4 cell count (aHR 0.87 ≥200 versus <200 cells/mm^3^; 95% CI 0.41–1.84; *P* 0.715), and WHO stage (aHR 1.83 for WHO stages I/II versus III/IV; 95% CI 0.83–4.01; *P* 0.133). Furthermore, we did not find association between renal impairment and arterial hypertension (aHR 1.19; 95% CI 0.55–2.61.59; *P* 0.657), TB diagnosis (aHR 2.04; 95% CI 0.75–5.55; *P* 0.161), total time on ART before the switch (per one year) (aHR 0.97; 95% CI 0.73–1.27; *P* 0.807) and calendar year of switch (aHR 0.83; <2015 versus ≥2015; 95% CI 0.34–2.00; *P* 0.674). The final multivariable model met the proportional hazard assumptions (Schoenfeld’s global *P* 0.5281).

**Table 3:** Univariate and multivariate Cox proportional hazards for predictors of renal impairment (with baseline covariates) (n = 556).

Variable	Univariate model Unadjusted HR (95% CI)	P value	Multivariate model Adjusted HR (95% CI)	P value
Age (per 10 years)	1.80 (1.41–2.31)	<0.001	2.22 (1.56–3.15)	<0.001
BMI (Kg/M^2^)				
≥18.5	1		1	
<18.5	1.12 (0.49–2.52)	0.791	0.96 (0.37–2.43)	0.924
CD4 count (cells/mm^3^)				
≥200	1		1	
<200	1.01 (0.54–1.90)	0.967	0.87 (0.41–0.84)	0.715
WHO stage				
I or II	1		1	
III or IV	1.74 (0.89–3.41)	0.107	1.83 (0.83–4.01)	0.133
Arterial hypertension				
No	1		1	
Yes	1.94 (0.97–3.90)	0.060	1.19 (0.55–2.61)	0.657
Tuberculosis diagnosis				
No	1		1	
Yes	3.08 (1.37–6.95)	0.007	2.04 (0.75–5.55)	0.161
Total time on ART before the switch (per one year)	0.98 (0.78–1.22)	0.842	0.97 (0.73–1.27)	0.807
Calendar year of switch				
<2015	1		1	
≥2015	0.78 (0.41–1.49)	0.450	0.83 (0.34–2.00)	0.674
First second-line ART				
Non-TDF–based	1		1	
TDF-based	1.87 (0.99–3.55)	0.055	2.12 (0.93–4.80)	0.072

Abbreviations: BMI, body mass index; ART, antiretroviral treatment; HR, hazard ratio; CI, confidence interval; WHO, World Health Organization; TDF, tenofovir disoproxil fumarate

### Confirmed CKD with 2 eGFR measurements 90 days apart

Out of 52 patients with renal impairment at the time of switch, 5 (9.6%) had a CKD confirmed by a second eGFR measurement <60 ml/min/1.73m^2^ at least three months apart. By the time of study closure, 26 participants (50%) were still on active follow-up, 15 participants (28.9%) were lost to follow-up, nine participants (17.3%) died and two participants (3.9%) transferred out to other clinic. Among the five participants with confirmed CKD at the time of switch, by the time of study closure, 2 participants were on active follow-up, 2 participants were lost to follow-up and 1 participant died.

Out of 41 patients with development of renal impairment after switch, 9 (22%) had a CKD confirmed by a second eGFR measurement <60 ml/min/1.73m^2^ at least three months apart. The median time to development of a confirmed CKD was 1.9 years (IQR 1.5–2.4). At the time of switch, 4 participants were prescribed abacavir (ABC), didanosine (ddl) and LPV/r, 4 participants were prescribed TDF, FTC, and LPV/r and 1 participant was prescribed TDF, FTC, and ATV/r combination. By the time of study closure, seven participants (17.1%) had at least one improved eGFR measurement ≥60 ml/min/1.73m^2^, thus most likely an AKI, one participant died and one participant still had eGFR <60 ml/min/1.73m^2^.

## Discussion

In this prospective cohort study in rural sSA, we found an incidence of renal impairment of 22 per 1,000 person-years (16.1–29.8) among participants under second-line treatment during a follow-up of 1,864.10 person-years. We found strong association between developing renal impairment and older age. The association between renal impairment and switch to TDF-based regimen was not statistically significant. At the time of switch–thus failure on first-line treatment–the prevalence of renal impairment was 7.6% and subsequently resolved in about half of these participants. Risk factors for renal impairment at the time of switch were older age, low BMI, arterial hypertension and total time on ART before the switch to second-line.

The comparison of renal impairment in different settings is challenging due to different laboratory methods for creatinine measurements, different definitions of renal impairment and different formula for calculation of eGFR [30]. A study reporting data from a community survey done in rural Kenya and Uganda reported a prevalence of CKD (defined as eGFR <60 mL/min/1.73m2 and/or proteinuria at a single measurement) in the general population of 6.8% with HIV infection being a risk factor (adjusted prevalence ratio 1.58 (1.11–2.24)) [31]. In a cross-sectional population study conducted in four African countries (South Africa, Burkina Faso, Kenya and Ghana) using the same definition [28] CKD was slightly higher (10.7%). A study from Tanzania [6] reported a CKD prevalence of 13.6% in elderly people (40–60 years) presenting to an outpatient clinic. In a previous study from our center—using the same definition as in this study—the prevalence of renal impairment among 1093 newly diagnosed PLWH was lower with 6.6% in patients under first-line treatment [9]. The increase to 7.6% from our current study could reflect increasing age of patients, selection of failing patients with higher risk of other comorbidities or possibly be drug-related. However, the previous two studies [6, 28], defined CKD using a single measurement of eGFR <60 mL/min/1.73m2 and/or proteinuria hence might lead to slight overestimation of CKD compared to our study [9].

Among PLWH treated with bPI-regimens from developed countries the risk of renal impairment and CKD is increased [14, 15]. In a study from the Data Collection on Adverse Events of Anti-HIV Drugs (D:A:D) CKD developed in 5.9% of patients on Atazanavir or Darunavir treatment (incidence rate, 10.0/1000 person-years (95% CI, 9.5–10.4/1000 person-years)) during a median follow-up of 6.8 years (5.4–7.1) [14]. This incidence is slightly lower compared to the results from our study, however, the authors defined CKD as having an eGFR <60 mL/min/1.73m2 on two measurements three months apart. In sSA, there are limited studies to evaluate the long term effects of bPIs on the kidney—mostly due to the lack of pharmacovigilance systems in the region [10, 11, 32]. This is of public health importance since the burden of CKD is on the rise in the region and it is associated with a high mortality and morbidity among PLWH [3, 4].

Age is a well-documented risk factor for renal impairment. In this study, the odds of having renal impairment at the time of switch to second-line increased by 55% for every 10 years increase in age. Studies from comparable settings have shown a similar association [6, 9, 33]. In our study, participants with a low BMI at the time of switch had almost 3 times higher odds of having renal impairment, which is likely explained by the fact that participants had advanced disease at the time of switch due to immunological or clinical treatment failure. Additionally, previous studies showed an increased risk of renal impairment and CKD among people with a low BMI [34]. This is a public health concern since low BMI is associated with high mortality among CKD patients [35]. Moreover, participants who were diagnosed with arterial hypertension had more than twice the odds of having CKD at the time of switch as previously reported [9, 29]. This is worrisome since patients with renal impairment have increased risk of developing cardiovascular diseases [36, 37]. Interestingly, each year of increase in ART use before the switch, reduced the risk of renal impairment by 22% at the time of switch. This could be due to the fact that more than half of our patients present at the clinic with advanced HIV disease and patients under longer-term treatment are usually more stable [9, 20, 21, 38].

The association between developing renal impairment and older age was confirmed also in patients developing renal impairment while being on a bPI as previously reported [6, 9]. At the same time, we observed an association between renal impairment and being switched to a bPI-regimen with a TDF-backbone versus a non-TDF backbone, although this was not statistically significant. The association of TDF and renal impairment is well known [12, 39, 40]. Nevertheless, this is an important finding since most treatment guidelines in sSA recommend the use of TDF- based regimens as both first-line and second-line treatments [10, 11]. The time point of switch to bPI-regimens reflected mostly immunological or clinical failure under first-line therapy and less virological failure (which usually precedes immunological failure), as routine HIV-1 RNA viral load testing was implemented only in 2018. The reason for raised creatinine levels therefore might have been concomitant diseases or an HIV-associated nephropathy. However, analyzing biobanked samples in retrospect in a previous study from the same cohort, we documented virological failure in less than half of patients (79/185 (42%)) being switched to second-line treatment based on immunological or clinical failure [41]. This could be due to the fact that fact before rollout of universal monitoring of HIV-1 RNA viral load, immunological or clinical failure was defined using CD4 cell count and WHO stage hence led to misclassification of patients with treatment failure [42].

This study highlights that renal impairment is of clinical relevance at the time of switch from first-line treatment, as well as after switch to bPI-regimens—thus signaling a clear need for i) improved awareness of kidney diseases in this setting; ii) more regular creatinine measurements; iii) improved history taking of concomitant drugs, and iv) developing improved pharmacovigilance activities.

In this study, we faced several limitations. Firstly, our definition of renal impairment is based on a single-point measurement of serum creatinine. Thus, we could not differentiate well between acute and chronic kidney failure for all patients due to lack of follow-up measurements. Despite recommendations by treatment guidelines, routine serum creatinine measurements are still not done in most of sSA due to logistic challenges, costs and shortages of reagents and machine-breakdowns. Also, patients face challenges to come to frequent visits due to high transport costs and daily responsibilities. Secondly, 22% of participants at the time of switch and almost 12% during follow-up had no serum creatinine measurements, which could represent a selection bias. As per guidelines patients who regularly visit the clinic would eventually receive a creatinine measurement with documentation. However, those not returning to the clinic could die from kidney failure. Thirdly, we did not systematically collect information on the use of traditional medicines, which is a potential differential reason for renal impairment. In a previous study, 70% of patients with CKD were reported to use traditional medicine in Tanzania [43]. Fourthly, we did not collect urine samples, therefore, information on proteinuria could not be evaluated in our study hence might lead to underestimation of the prevalence of renal impairment among our patients. Finally, since we conducted an observational study, there may be unmeasured confounders that we are unable to account for hence may have affected our results.

## Conclusion

In sSA, PLWH who develop treatment or clinical failure have limited ART options for the second-line treatment. Our study shows a high prevalence of renal impairment at the time of switch from first-line ART to bPI and a high incidence of renal impairment after switch to bPI-regimens. Additionally, our findings support strong associations between renal impairment and older age, BMI, arterial hypertension and time on ART before the switch. Given the need for lifelong ART, these data support the implementation of universal routine monitoring of renal function, the need of follow-up measurements improved pharmacovigilance monitoring.

## Supporting information

S1 Table Demographic characteristics of participants included in follow up. Abbreviations: eGFR, estimated glomerular filtration rate; BMI, body mass index; WHO, World Health Organization; IQR, inter quartile range; ART, antiretroviral treatment; AZT, zidovudine; 3TC, lamivudine; NVP, nevirapine; EFV, efavirenz; TDF, tenofovir disoproxil fumarate; FTC, emtricitabine; ABC, abacavir; LPV/r, lopinavir/ritonavir; ATV/r, atazanavir/ritonavir; ddI, didanosine. *requested only in patients with suspected treatment failure and those who were involved in s specific study.
(DOCX)

S1 File Minimal KIULARCO dataset. (XLS)
