# Supplementary material for: Prevalence, incidence and predictors of renal impairment in persons with HIV receiving protease-inhibitors in rural Tanzania
Source: PLoS One. 2021 Dec 15;16(12):e0261367. doi: 10.1371/journal.pone.0261367 (PMC8673654; doi:10.1371/journal.pone.0261367)
Supplement: S1 Table — Abbreviations: eGFR, estimated glomerular filtration rate; BMI, body mass index; WHO, World Health Organization; IQR, inter quartile range; ART, antiretroviral treatment; AZT, zidovudine; 3TC, lamivudine; NVP, nevirapine; EFV, efavirenz; TDF, tenofovir disoproxil fumarate; FTC, emtricitabine; ABC, abacavir; LPV/r, lopinavir/ritonavir; ATV/r, atazanavir/ritonavir; ddI, didanosine. *requested only in patients with suspected treatment failure and those who were involved in s specific study. (DOCX) [file pone.0261367.s001.docx]

**S1 Table: Demographic characteristics of participants included in follow up**

|  | Total cohort  Absolute number (%)  n=556 | Normal kidney function at baseline (eGFR ≥60 ml/min/1.73m^2^)  n=515 | Renal impairment during follow-up (eGFR <60 ml/min/1.73m^2^) n=52 |
| --- | --- | --- | --- |
| Sex |  |  |  |
| Male | 205 (36.9) | 194 (37.7) | 11 (26.8) |
| Female | 351 (63.1) | 321 (62.3) | 30 (73.2 ) |
| Age , years, Median (IQR) | 40.8 (33.0 – 48.3) | 40.6 (32.4 – 47.4) | 48.9 (38.0 – 58.4) |
| WHO Stag e |  |  |  |
| I | 106 (19.1) | 101 (19.6) | 5 (12.2) |
| II | 119 (21.4) | 112 (21.8 ) | 7 (17.1) |
| III | 195 (35.1) | 175 (34.0) | 20 (48.8) |
| IV | 134 (24.1) | 125 (24.3) | 9 (22.0) |
| Missing | 2 (0.4) | 2 (0.4) | 0 (0) |
| BMI (Kg/M^2^) |  |  |  |
| <18.5 | 82 (14.8) | 75 (14.6) | 7 (17.1) |
| ≥18.5 – 24.9 | 302 (54.3) | 277 (53.8) | 25 (61.0) |
| ≥25 | 113 (20.3) | 105 (20.4) | 8 (19.5) |
| Missing | 59 (10.6) | 58 (11.3) | 1 (2.4) |
| CD4 count (cells/mm3) |  |  |  |
| <200 | 230 (41.4) | 213 (41.4) | 17 (41.5) |
| ≥200 | 307 (55.2) | 284 (55.2) | 23 (56.1) |
| Missing | 19 (3.4) | 18 (3.5) | 1 (2.4) |
| Tuberculosis |  |  |  |
| Yes | 41 (7.4) | 34 (6.6) | 7 (17.1) |
| No | 514 (92.5) | 480 93.2) | 34 (82.9) |
| Missing | 1 (0.2) | 1 (0.2) |  |
| Arterial hypertension |  |  |  |
| Yes | 91 (16.4) | 80 (15.5) | 11 (26.8) |
| No | 461 (82.9) | 432 (83.9) | 29 (70.7) |
| Missing | 4 (0.7) | 3 (0.6) | 1 (2.4) |
| First ART regimen after switch |  |  |  |
| ABC+ddI+LPV/r | 46 (8.3) | 39 (7.6) | 7 (17.1) |
| TDF+3TC+LPV/r | 3 (0.5) | 3 (0.6) | 0 (0) |
| TDF+FTC+LPV/r | 113 (20.3) | 102 (19.8) | 11 (26.8) |
| ABC+3TC+LPV/r | 9 (1.6) | 8 (1.5) | 1 (2.4) |
| AZT+3TC+LPV/r | 31 (5.6) | 29 (5.6) | 2 (4.9) |
| AZT+3TC+ATV/r | 120 (21.6) | 115 (22.3) | 5 (12.2) |
| TDF+ABC+LPV/r | 1 (0.2) | 1 (0.2) | 0 (0) |
| TDF+FTC+ATV/r | 161 (29.0) | 151 (29.3) | 10 (24.4) |
| TDF+3TC+ATV/r | 3 (0.5) | 3 (0.6) | 0 (0) |
| ABC+3TC+ATV/r | 24 (4.3) | 22 (4.3) | 2 (4.9) |
| Other second-line | 45 (8.1) | 42 (8.2) | 3 (7.3) |
| Viral load at time of swtich*  Copies/ml |  |  |  |
| <1000 | 121 (21.8) | 112 (21.8) | 9 (22.0) |
| ≥1000 | 193 (34.7) | 187 (36.3) | 6 (14.6) |
| Missing | 242 (43.5) | 216 (41.9) | 26 (63.4) |
| Time under observation after switch (years) |  |  |  |
| Median (IQR) | 3.5 (1.6 – 5.1) | 3.8 (2.0 – 5.1) | 0.24 (0.1 – 0.5) |
| Calendar year of switch |  |  |  |
| <2015 | 330 (59.4) | 303 (58.8) | 27 (65.8) |
| ≥2015 | 226 (40.6) | 212 (41.2) | 14 (34.2) |

*Abbreviations:eGFR, estimated glomerular filtration rate; BMI, body mass index; WHO, World Health Organization; IQR, inter quartile range; ART, antiretroviral treatment; AZT, zidovudine; 3TC, lamivudine; NVP, nevirapine; EFV, efavirenz; TDF, tenofovir disoproxil fumarate; FTC, emtricitabine; ABC, abacavir; LPV/r, lopinavir/ritonavir; ATV/r, atazanavir/ritonavir; ddI, didanosine*

**requested only in patients with suspected treatment failure and those who were involved in s specific study*
